# The Effect of Shear Deformation on C-N Structure under Pressure up to 80 GPa

**DOI:** 10.3390/nano11040828

**Published:** 2021-03-24

**Authors:** Valentin Churkin, Boris Kulnitskiy, Pavel Zinin, Vladimir Blank, Mikhail Popov

**Affiliations:** 1 Technological Institute for Superhard and Novel Carbon Materials, Centralnaya Str. 7a, Troitsk, 142190 Moscow, Russia; churkin_valentin@rambler.ru (V.C.); boris@tisnum.ru (B.K.); vblank@tisnum.ru (V.B.); 2 Moscow Institute of Physics and Technology State University, Institutskiy per. 9, Dolgoprudny, 141700 Moscow, Russia; 3 Scientific and Technological Center of Unique Instrumentation, Russian Academy of Sciences, Butlerova str. 15, 117342 Moscow, Russia; 4 Research Centre, National University of Science and Technology MISiS, Leninskiy prospekt 4, 119049 Moscow, Russia

**Keywords:** *g*-C_3_N_4_, high pressure, phase diagram, onions

## Abstract

We studythe effect of shear deformation on graphitic *g*-C_3_N_4_ under pressures of up to 80 GPa at room temperature. *g*-C_3_N_4_ samples are transformed from initial amorphous flakes into onion-like structures, in which the nitrogen content in the quenched samples decreases with increasing pressure (from 42% in the initial conditions to 1% at 80 GPa). The concentration of the *sp*^2^ bonds also decreases from 1 (the initial sample) to 0.62 with increasing pressure to 80 GPa. This transformation of the sample is due to the fact that in the pressure range of 55–115 GPa, the equilibrium phase is not a diamond, but instead, carbon onions cross-linked by *sp*^3^ bonds, which are denser than diamonds. The results of our study show that the presence of nitrogen in *sp*^3^-bonded structures at pressures of higher than 55 GPa reduces the density and, accordingly, carbon structures without nitrogen become thermodynamically favorable.

## 1. Introduction

According to recent experimental and computer modeling studies, fullerene-type onions cross-linked by *sp*^3^ bonds are the equilibrium phase of the carbon phase diagram at 55–115 GPa [1,2]. This discovery allows us to re-evaluate the phase diagram of C-N compounds from the perspective of obtaining new nanocluster-based materials. For many years, intense interest in the synthesis of new superhard materials has been generated by predictions on the unusual properties of saturated, i.e., *sp*^3^-hybridized, crystalline C_3_N_4_-phases [3]. Numerous attempts to obtain hypothetical phases have been undertaken since the Liu and Cohen publication. Despite many claims of making new CN_x_ films and C_3_N_4_ crystallites, no convincing evidence has yet been obtained for the dense C_3_N_4_ phases, other than graphite-like phases [4,5]. However, the synthesis of several new C-N-based phases [6,7,8,9,10,11], conducted recently, demonstrates how little is known about the behavior of C-N compounds under high pressure and indicates that the search for new high-pressure phases should be continued. Horvath-Bordon et al. [6] reported on high-pressure synthesis of a well-crystallized compound C_2_N_2_(NH) with an N:C ratio of 3:2, in which all of the carbon atoms are tetrahedrally coordinated. The presence of structural hydrogen atoms was recognized (by nanoSIMS) and the composition of C2N2(NH) was determined. The crystal structure of the new compound (defect- wurtzite *dwur*-C_2_N_2_(NH)) was determined by combining electron-diffraction results with primary principle theoretical studies. This finding was confirmed by Salamat et al. [12]. The crystal structure of carbon nitride under high pressure and temperature was investigated up to megabar pressures, using graphitic C_3_N_4_(g-C_3_N_4_) as a starting material. It transformed to an orthorhombic phase above 30 GPa and 1600 K, which has similar unit cell parameters to those of reported hydrogen-bearing carbon nitride phases, such as C_2_N_2_(NH) and C_2_N_2_(CH_2_) [8]. These results suggest that in the studied wide pressure and temperature range, hydrogen-bearing carbon nitride favors an orthorhombic structure with a fundamental composition of C_2_N_2_X, where X = NH or CH_2_. Another problem in achieving phase transition in a C_3_N_4_ system relates to the fact that g-C_3_N_4_ becomes transparent at high pressures [9,13] and, therefore, is difficult to heat by means of a laser to overcome the reaction activation barrier. For example, under hydrostatic conditions, graphitedoes not transform to diamond at pressures of less than 80 GPa [14], while activation of the phase transition by shear deformation leads tothe direct transformation of graphite to diamond at pressures of17–19 GPa at room temperature [1,2,15].

However, there is a way to overcome the reaction activation barrier by applying shear stress under high pressure [16,17]. In the present study, we investigate the effect of shear stress on the behavior of the C-N phase under high pressure, in the shear DAC (SDAC). Features of the activation of phase transitions in carbon nanocluster-based materials using SDAC were discussed in Reference [18].

## 2. Materials and Methods

Raman spectra were recorded with a Renishaw inVia Raman microscope (excitation wavelength 532 nm) (Renishaw plc, Spectroscopy Products Division, New Mills, Wotton-under-Edge, Gloucestershire GL12 8JR, United Kingdom). A high-resolution transmission electron microscopy (HRTEM) study was performed using a JEM 2010 TEM microscope (JEOL Ltd. 3-1-2 Musashino, Akishima, Tokyo 196-8558, Japan)with a GIF Quantum attachment for EELS. (DigitalMicrograph, Gatan’s software, GATAN: 5794 W. Las Positas Blvd. Pleasanton, CA 94588, United States) for transmission electron microscopy (TEM), was used in the present study. The ratio of the element concentrations was determined usingthe ratio of the corresponding areas on the spectrum. The specimen was extruded using a needle from the gasket on the slide plate, broken into fragments, and then deposited on the grid. X-ray diffraction data (XRD) were obtained using an Empyrean PANalytical powder diffractometer (Malvern Panalytical B.V., De Schakel 18, Kamer 50, Eindhoven, 5651 GH, The Netherlands) equipped with a PIXcel3D detector (Bragg-Brentano geometry, CuK_α_radiation, step size 0.0131°). For the monochromatization of the primary beam and high intensity of the incident X-ray beam on the sample, we used a Bragg–BrentanoHD X-ray optics module (multilayer paraboloid mirror that provided separation of K_β_ radiation and gave the superposition of K_α1_ and K_α2_).

The graphitic-like phase, *g*-C_3_N_4_, was prepared via a solid-state reaction between cyanuric chloride or its fluoro-analog and lithium nitride [19]:

C_3_N_3_Cl_3_+3Li_3_N→*g*-C_3_N_4_+3LiCl

**Figure 1:**
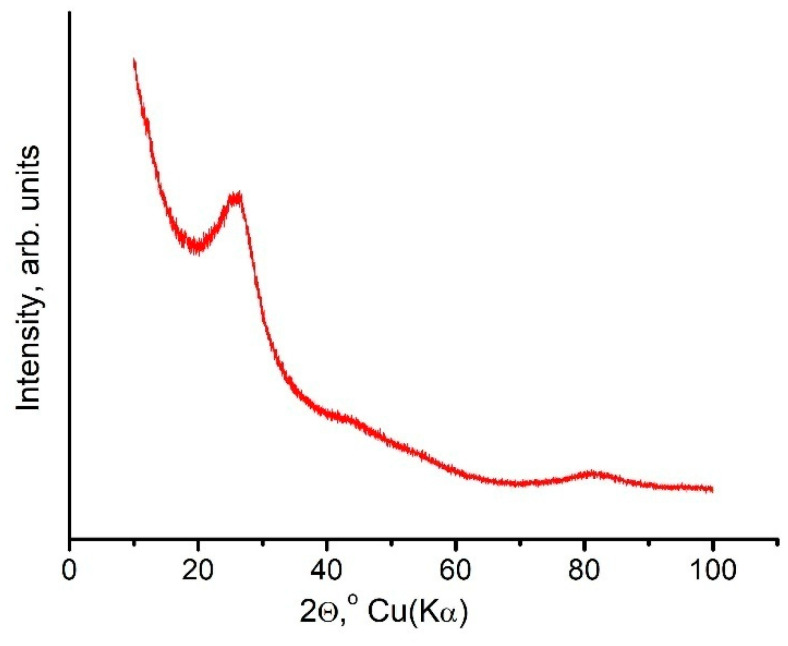
The X-ray diffraction pattern of the starting *g*-C_3_N_4_ material.

The spherical modification of carbon nitride was obtained when added to a reactor, as a substrate, with nano-size silica spheres, using the procedure described previously [20]. The X-ray diffraction pattern of the starting *g*-C_3_N_4_ material is shown in Figure 1. The initial graphite-like *g*-C_3_N_4_ is a partially three-dimensional ordered structure, with the following parameters: a = 0.2370 ± 0.0005 nm and c = 0.686 ± 0.025 nm, which corresponds to the C-N bond length equal to L = 0.137 nm. This corresponds to the known data described inthe literature [4,5,19,21].

According to EELS analysis, the nitrogen concentration in the starting material varied from 23 to 43 at. %. Some lack of nitrogen may be due to the fact that nitrogen deficiency is common in carbon nitrides because of the stability of the easily-formed N_2_ molecules [21].

The *g*-C_3_N_4_ powder was placed in a 60 μm hole of a pre-pressed tungsten gasket without any pressure transmitting media. Pressure was measured from the stress-induced shifts of Raman spectra from a diamond anvil tip [22]. Before loading the g-C_3_N_4_ specimen, it was kept in a vacuum at a temperature of 95 °C for two hours.Three experiments were conducted with different initial pressure loading levels: (a) 20 GPa; (b) 53 GPa; and (c) 70 GPa. On achieving an initial pressure, shear deformation was applied to the sample by means of rotating one of the anvils around the load axis [17]. At each initial pressure, the anvil was rotated by 20° and returned to its initial position. After applying shear deformation, the pressure increased in the sample to 25, 57, and 79 GPa, accordingly. Pressure increase was induced by a jump in the elastic module and volume [17]. After removing the pressure and extracting the gasket from the diamond cell, the obtained material was examined.

## 3. Results

### 3.1. TEM Study

**Figure 2:**
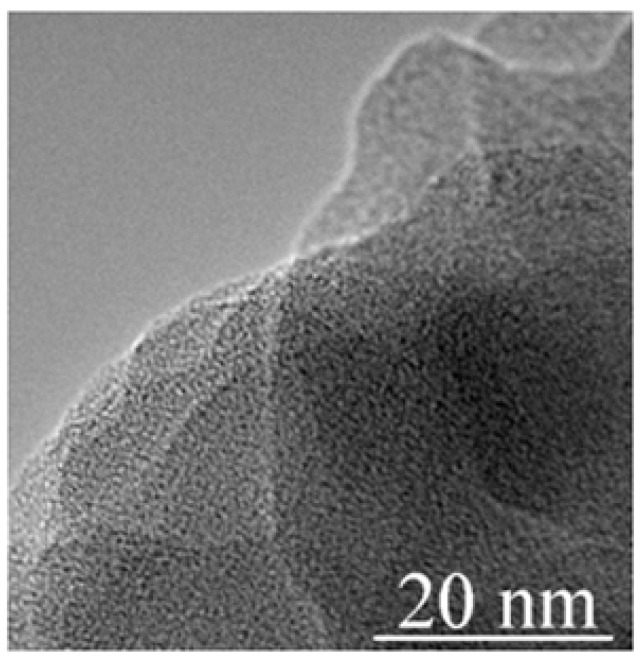
TEM image of the *g*-C_3_N_4_ specimen before high-pressure treatment. The specimen resembles a set of amorphous flakes.

A TEM image of the starting *g*-C_3_N_4_ material is shown in Figure 2. The specimen resembles a cluster of amorphous flakes.

**Figure 3:**
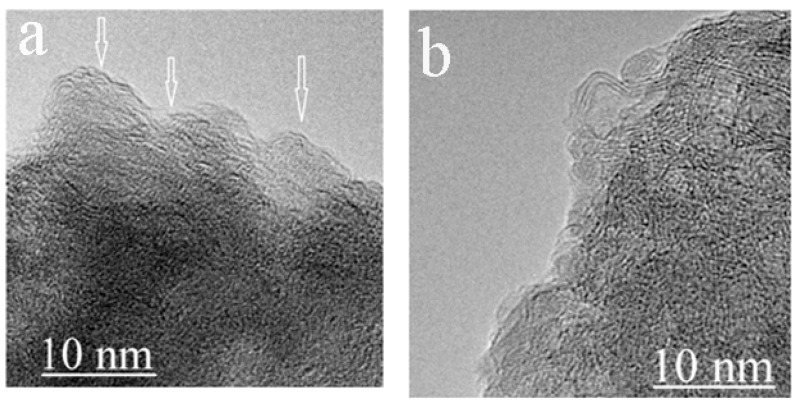
TEM images of the *g*-C_3_N_4_ specimen after shear deformation at 20–25 GPa; (**a**) an image with graphene-like layers shown by arrows. (**b**) An image showing an onion-like structure of atypical size.

After shear deformation at 20–25 GPa, the morphology of the specimenchanges. Onion-like structures start to form at the edges of the flakes; their size can be estimated from the TEM image shown in Figure 3 (~5 nm).

Table 1 shows the N content after shear deformation is applied at different pressures. We see that after shear deformation, the nitrogen content decreases. Moreover, the higher the pressure before shear deformation, the lower the nitrogen content after deformation. Onion-like structures are also seen in the specimen after being subjected to high shear deformation at 53 GPa.

**Figure 4:**
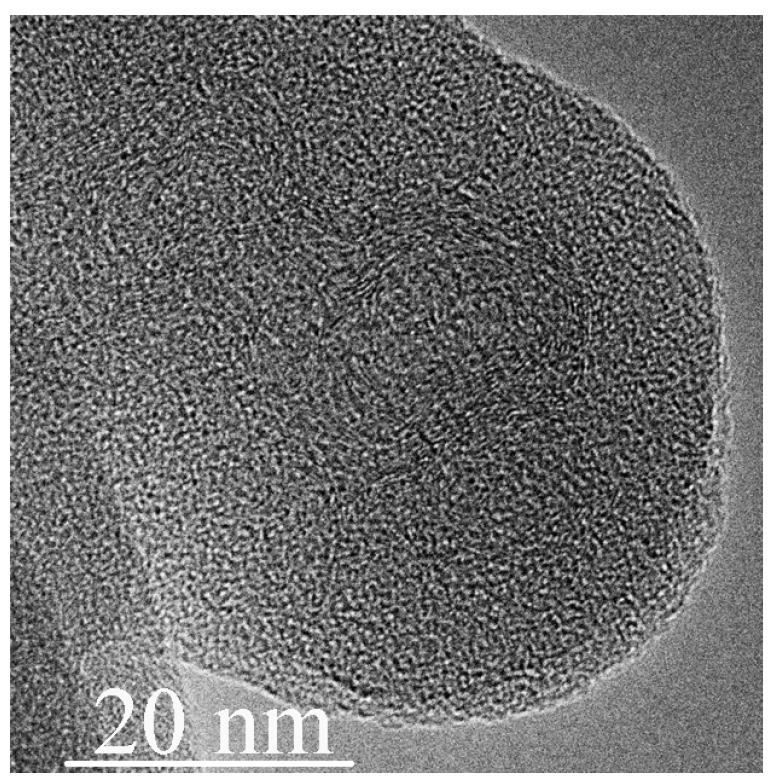
TEM image of the *g*-C_3_N_4_ specimen after shear deformation at 53–57 GPa.

The TEM image of the specimen, after quenching from 57 GPa, is shown in Figure 4. The size of the onion-like fragments increases to 20 nm. Analysis of the EELS spectra demonstrates that the nitrogen content drops to 7% from 42% (Table 1).

**Figure 5:**
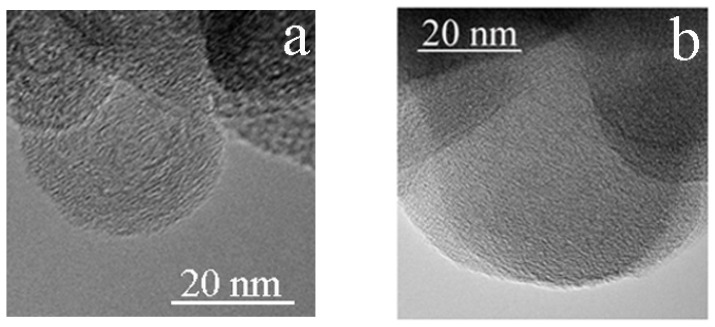
TEM images of different onions (**a**,**b**), obtained after shear deformations of the g-C3N4 specimen at 70–79 GPa.

Figure 5 shows the TEM images of the specimen quenched from 79 GPa after shear deformation at 70 GPa.

Onion-like structures are seen in the specimen after shear deformation at pressures of 70–79 GPa (Figure 5). The average size of the onion-like structures is still around 20 nm. However, the nitrogen content drops drastically to 1% (Table 1). These data indicate that *sp*^3^-bonded carbon nitride cannot be obtained at 70 GPa under heavy shear deformation.

**Figure 6:**
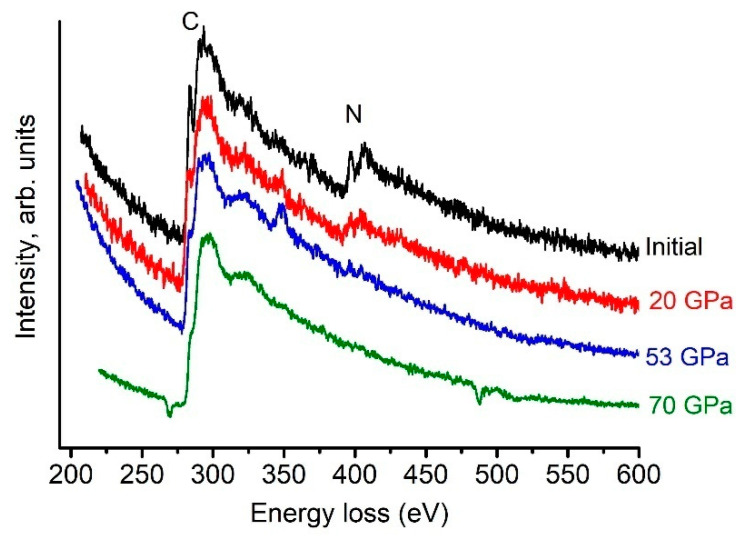
EELS spectra measured on quenched C-N phases. The exposure time when receiving the EELS spectrum is a few seconds. The particle size is several nanometers.

Three different types of nonoxidized N atoms can be found in C-N materials [23]: (a) “pyridinic”, (b) “pyrrolic”, and (c) “graphitic” nitrogen, with binding energies of ≈399.0, ≈400.3, and ≈401–403 eV, respectively. As follows from the EELS spectra (Figure 6), “graphitic” nitrogen is detected in the C-N phases before and after shear deformation. The presence of strong C-K and N-K π* peaks indicates that the starting g-C_3_N_4_ phase is preferentially *sp*^2^ bonded [24,25]. To estimate the concentration of *sp*^2^ bonds in the carbon phase, we used the method described in [26]; the results are shown in Table 2. A detailed review of the methods developed for treating the EEELs spectra of carbon can be found elsewhere [27].

### 3.2. Raman Study

**Figure 7:**
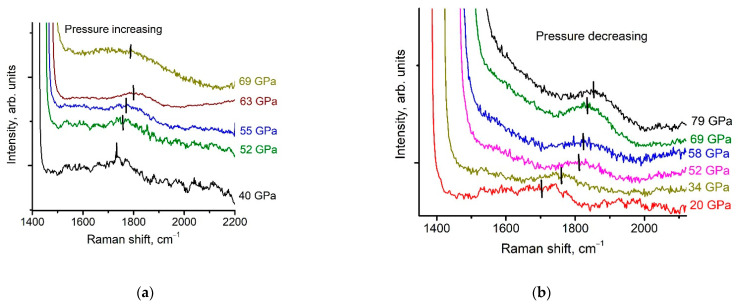
The behavior of the Raman spectra of a *g*-C_3_N_4_ specimen as a function of pressure: (**a**) increasing pressure, (**b**) decreasing pressure after shear at 70 GPa.

**Figure 8:**
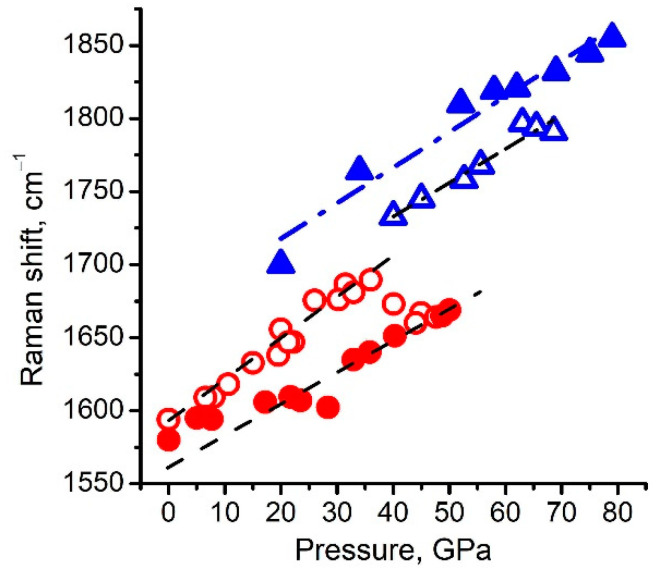
Position of the Raman peak of the 1620 cm^−1^peak of the *g*-C_3_N_4_ specimen as a function of pressure: filled triangles for peaks after shear deformation; open triangles for peaks before shear deformation at 70 GPa. Center of the Raman peak of the 1600 cm^−1^peak of carbon onions with a diameter of ~20 nm from Reference [30]; open circles for peaks before deformation; filled circles for peaks after shear deformation.

Strong fluorescence makes it difficult to measure the Raman spectra of the C-N phases at low pressures [28]. However, abroad band on the Raman spectra can be detected at pressures above 40 GPa. It is centered at 1730 cm^−1^ at 40 GPa (Figure 7a). We assume that the position of this peak coincides with the 1620 cm^−1^peak at an ambient pressure measured in [28] using UV laser. The position of the 1620 cm^−1^peak as the function of pressure is shown in Figure 8. The peak positions were found using OriginPro 8 (OriginLab Corporation) tools (OriginLab Corporation, One Roundhouse Plaza, Suite 303, Northampton, MA 01060, United States). The positions are marked in Figure 7. Errors correspond to the symbol size in Figure 8.

We denote these curves as ν_g_(*P*) curves. Above 40 GPa, the pressure dependence of the 1620 cm^−1^peak position can be considered as linear as pressure increases:

ν_g_ = 1669(13) + 2.432(0.22) × *P* (1)

It is interesting to compare this behavior with that of graphite above 35 GPa: ν_a_ = 1669(4) + 0.41(0.06) × *P* [29]. The difference in slope shape indicates that the graphitic phase is more rigid above 40 GPa. We explain such a difference in rigidity with the fact that graphite transforms into onion-like structures [2,30], whereas the C-N ring keeps its structure in the g-C_3_N_4_ phase. Figure 7b shows that it is possible to trace the 1620 cm^−1^peak until 20 GPa, after the application of shear deformation at 70 GPa.

Below 76 GPa, the pressure dependence of the 1620 cm^−1^peak position can be considered as linear, while pressure decreases:

ν_g_ = 1640(14) + 2.32(0.24) × *P* (2)

Fluorescence starts growing with decreasing pressure below 20 GPa, and the 1620 cm^−1^peak becomes undetectable. As we can see, the slope of the line has not been changed.

Transformation of the *g*-C_3_N_4_ into 20 nm onion-like structures with low nitrogen contents after shear deformation, at a pressure or 70 GPa, makes it interesting to compare the dependence of the peak at ~1600 cm^−1^ on pressure for the C-N sample withthat of 20 nm pure carbon onions.Carbon onions with a diameter of ~20 nm were synthesized from natural gas by means of non-catalytic partial oxidation [30].

## 4. Discussion

One of the main results of this study is that, under shear deformation at high pressures, nitrogen runs away from the carbon structure. Moreover, the higher the pressure, the higher the loss of nitrogen. High-pressure measurements on the graphitic C_3_N_4_ (g-C_3_N_4_) phase, using Brillouin light scattering (BLS) up to 41.5 GPa and X-ray Raman scattering (XRS) up to 26 GPa, reveal no structural phase transition and, unlike graphite, no *sp*^2^ to *sp*^3^rehybridization in this pressure range. It indicates that the loss of nitrogen occurs during shear deformation, which is unexpected as high pressure should make the diffusion of nitrogen atomsdifficult. We do not know where the nitrogen atoms go; it is possible that shear deformation leads to the formation ofthe so-called atomic phase of nitrogen, which is stable under 70 GPa [31] or N_2_ molecules [32]. The severe loss of nitrogen found in this study is not in line with the theoretical predictions [33]. It was predicted that several CN, CN_2,_ and C_3_N_4_ phases would be thermodynamically stable at the pressure range of 14–98 GPa. The behavior of the nitrogen content under high pressure has beenstudied by several groups. The synthesis of amorphous *sp*^2^-bonded carbon nitrides at high pressure, from carbon and nitrogen tetracyanoethylene in a DAC at 2000 °C, showed that the amount of nitrogen incorporated into the network increases with pressure, ranging from 24% (C_3_N) at 18 GPa to 38% at 42 GPa (C_3_N_1.9_) [24]. Heating turbostratic carbon nitride in the 4.7–17.8 GPa range has revealed the decomposition of t-CN at high temperatures [34]. The onset temperature of t-CN decomposition increases from 990(10) to 1850(50) K. Decomposition results in the formation of disordered graphite at pressures below 8 GPa, and diamond at higher pressures. In our study, we did not observe the formation of the highly incompressible Pnnm CN compound with *sp*^3^-hybridized carbon, as was synthesized above 55 GPa and 7000 K [10].

Another important result of this study is the first observation of the C-N onion-like structure under pressure. Measurements of the Raman spectrum behavior of the C-N onions under pressure show it to be similar to that of pure carbon onions. To conduct a comparison of the behavior of the carbon onions under pressure with that of the C-N onion-like structure, we loaded the carbon onions into a diamond chamber, following the same procedure as for the C-N sample. Shear deformation was applied at a pressure of 45 GPa (Figure 8). The pressure dependence of the 1600 cm^−1^peak position before shear deformation can be considered to be linear as pressure increases:

ν_g_ = 1605(7) + 1.80(0.26) × *P* (3)

After shear deformation, the pressure dependence of the 1600 cm^−^^1^peak position can be also considered as linear as pressure decreases:

ν_g_ = 1576(5) + 1.74(0.17) × *P* (4)

The abrupt decrease in the frequency of the observed Raman peak from 1605 cm^−1^ to 1576 cm^−1^ under pressure in carbon onions, is associated with the formation of an interlayer with *sp*^3^ bonds [30]. We assume that the shift of the 1600 cm^−1^peak line in the C-N system is also associated with the formation of the C-N onion-like structure.

Apparently, the partial transformation of *sp*^3^to *sp*^2^ bonds at ~15 GPa during unloading, causes a “jump” in Raman frequency with one linear dependency (1560 cm^−1^ at 0 GPa) to another (1590 cm^−1^ at 0 GPa) (Figure 7). After the pressure releases, 50% of the *sp*^3^ bonds remain in the samples, according to XPS data [30]. In the Raman spectra, along with the D and G peaks (*sp*^2^ bonds), there is a 1560 cm^−1^ band (*sp*^3^ bonds). A similar transition of *sp*^2^ to *sp*^3^ hybridization at pressures above 15–20 GPa, and a partial reverse transition when the pressure is released, were also observed in other carbon materials [35,36,37,38,39].

In the case of the C-N sample, the dependence of the Raman band ~1600 cm^−1^ on pressure was almost the same as for carbon onions. However, up to the maximum pressure in our experiments of 79 GPa, there were no noticeable deviations from linear dependence. At the same time, with increasing pressure, the nitrogen content decreased from 35% to 1% (Table 1). When unloading at a pressure of about 55 GPa, a jump to higher frequencies was observed, as in the case of carbon onions at ~15 GPa.

This difference in the behavior of carbon onions and the onion-like structure formed from C-N material athigh pressure can be explained using a novel carbon phase diagram [2]. The diagram contains an experimentally revealed zone of diamond instability in the 55–115 GPa pressure range, while at room temperature. Diamond formation stops at these pressures, while the already formed diamonds turn into carbon onions cross-linked by *sp*^3^ bonds. This zone is consistent with the model (basedon atomistic modeling) that describes the possible nanostructures as denser than diamond in the 55–100 GPa pressure range [1,2,40]. The results of our study show that the presence of nitrogen in *sp*^3^-bonded structures, at pressures higher than 55 GPa, reduces the density and, accordingly, carbon structures without nitrogen become thermodynamically favorable. Indeed, the volume of onions cross-linked by *sp*^3^ bonds under a pressure of 70 GPa is 4.8 Å^3^/atom [2]. The atomic phase of nitrogen, which is stable under a pressure of 70 GPa [31], has a volume of 5.8 Å^3^/atom [41]. A hypothetical compound, β-C_3_N_4_ [3,42], has avolume of 6.2 Å^3^/atom under ambient conditions. The extrapolation of this value to a pressure of 70 GPa, by analogy with diamond compressibility [42], yields a volume of 6.0 Å^3^/atom. Consequently, in the case of possible pressure-induced decomposition of β-C_3_N_4_ (7 atoms, the volume is 7atoms × 6.0Å^3^/atom = 42 Å^3^), we will observe carbon onions (3 atoms, 3atoms × 4.8Å^3^/atom = 14.4 Å^3^) and the atomic phase of nitrogen (4 atoms, 4atoms × 5.8Å^3^/atom = 23.7 Å^3^). The total volume of the decomposed phases is 38.1 Å^3^, which is less than that of β-C_3_N_4_-42 Å^3^.

## 5. Conclusions

The effect of pressure and shear deformation on amorphous C_3_N_4_ at room temperature leads to the formation of onion-like structures, in which the nitrogen content decreases with increasing pressure (from 42% in the initial sample to 1% in the sample after 80 GPa).

The concentration of *sp*^2^ bonds also decreases from 1 (the initial sample) to 0.62, with increasing pressure to 80 GPa.

The presence of nitrogen in *sp*^3^-bonded structures at pressures higher than 55 GPa reduces the density and, accordingly, carbon structures without nitrogen become thermodynamically favorable.

## Figures and Tables

**Table 1:** Concentrations of carbon and nitrogen in the *g*-C_3_N_4_ specimen after shear deformation.

Sample	C, at. %	N, at. %
Initial	58	42
20–25 GPa shear	76	24
53–57 GPa shear	93	7
70–79 GPa shear	99	~1

**Table 2:** The *sp*^2^ content using the approach described in Reference [26]. The area under the *sp*^2^ peak (280–285 eV) was divided by the value of the area of the *sp*^3^ peak (286–292 eV). As we can see, the concentration of nitrogen decreases, as well as the concentration of the *sp*^2^ bonds, with increasing pressure in the quenched specimens.

Sample	*sp*^2^ Content
Initial	1
20–25 GPa shear	0.92
53–57 GPa shear	0.77
70–79 GPa shear	0.62

## Data Availability

The data presented in this study are available on request from the corresponding author.
